# Nodular fasciitis of the breast clinically resembling breast cancer in an elderly woman: a case report

**DOI:** 10.1186/s13256-017-1219-1

**Published:** 2017-03-03

**Authors:** Satoshi Hayashi, Shunsuke Yasuda, Nana Takahashi, Satoshi Okazaki, Kei Ishibashi, Masahiro Kitada, Naoyuki Miyokawa

**Affiliations:** 1 Breast Center, Asahikawa Medical University Hospital, Midorigaoka-Higashi 2-1-1-1, Asahikawa, Hokkaido 078-8510 Japan; 2 Department of Pathology, Asahikawa Medical University Hospital, Midorigaoka-Higashi 2-1-1-1, Asahikawa, Hokkaido 078-8510 Japan

**Keywords:** Nodular fasciitis, Breast, Benign, Elderly woman, Immunostaining

## Abstract

**Background:**

Nodular fasciitis is a benign reactive proliferative lesion of fibroblast cells, which can occur throughout the body. However, it has rarely been reported in the breast of an elderly woman.

**Case presentation:**

Our patient was an 88-year-old Asian woman who had noticed a mass in her right breast for 1 month before presentation to our hospital. The mass was elastic-hard and 20 mm in size. No qualitative diagnosis was made by core needle biopsy. Because of potentially malignant findings on mammography and ultrasonography, she underwent an excisional biopsy. Microscopically, spindle cell proliferation with abundant elastic fibers were observed. The tumor cells were positive for α-smooth muscle actin and negative for pancytokeratin, β-catenin, and cluster of differentiation 34. Based on these morphological and immunohistochemical features, a diagnosis of nodular fasciitis was made. All resection margins in the specimen were tumor-free. The patient has been disease-free for over 12 months.

**Conclusions:**

Nodular fasciitis shows clinical features and imaging findings similar to those of breast cancer. To avoid unnecessary surgery, nodular fasciitis should be taken into consideration when there is spindle cell proliferation found by biopsy.

## Background

Although nodular fasciitis (NF) of the breast appears malignant on clinical and diagnostic imaging studies, it is difficult to diagnose by cytology or needle biopsy, as well as to treat. NF rarely occurs in the breast and is, furthermore, most frequently reported in relatively young people. In this report, we present a rare case of an elderly woman with NF of the breast. Confusion with this pathology and neoplasm can be very dramatic; pathologists must be very aware of this to make a correct diagnosis of cancer.

## Case presentation

**Fig. 1:**
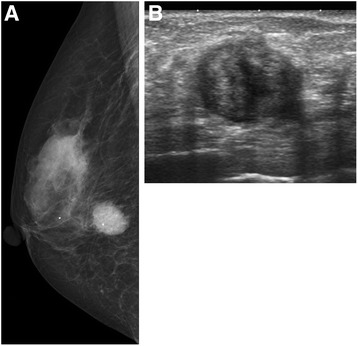
Imaging findings. **a** Mammogram shows a round, microlobulated mass. **b** Ultrasonogram shows a hypoechoic solid mass with an irregular margin

**Fig. 2:**
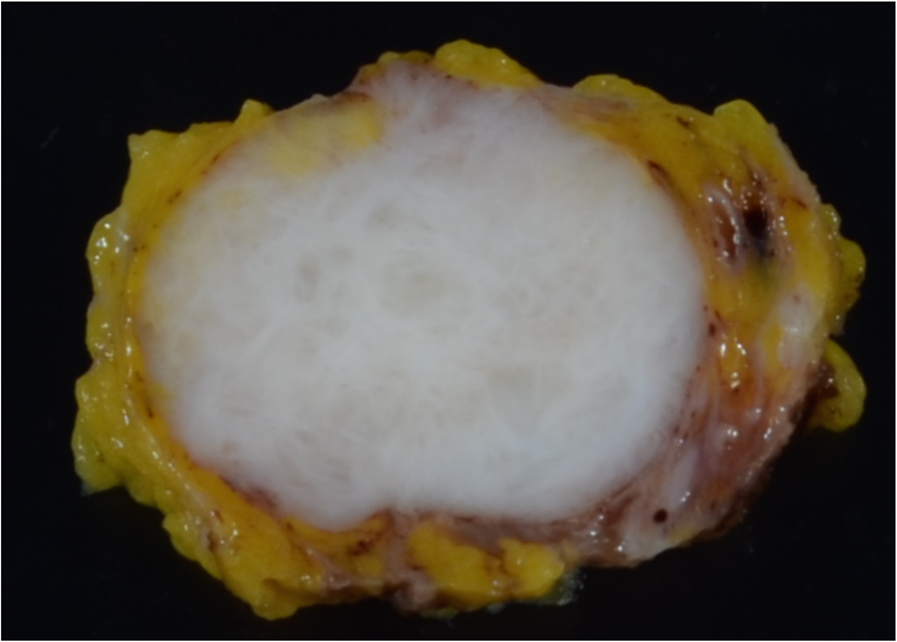
Macroscopic appearance of the resected tumor. The tumor appeared solid and milky white, and the tumor was clearly demarcated from the surrounding adipose tissue

**Fig. 3:**
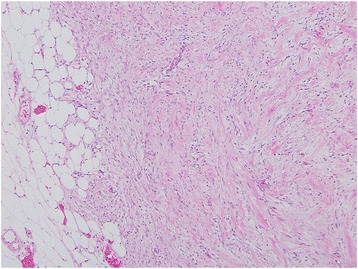
Pathological findings (original magnification ×200). The tumor was composed of spindle cells with abundant elastic fiber and had irregular infiltrative margins partially extending into the adipose tissue

Our patient was an 88-year-old Asian woman who had noticed a mass in her right breast 1 month earlier. She visited our department, where she had been receiving outpatient treatment for other conditions. In the lower outer quadrant of the right breast, a less mobile, elastic-hard mass measuring 20 mm in diameter was detected 25 mm from the nipple. Neither the axillary nor the supraclavicular lymph nodes were palpable. Regarding the patient’s medical history, she had been followed at our department for 12 years after surgery for left breast cancer (T1N0M0, hormone receptor-negative, no postoperative therapy); however, she had no history of apparent trauma to the breasts. Mammography revealed a dense, round, microlobulated mass (Fig. 1a). Ultrasonography of the breast revealed a round, hypoechoic mass measuring 18.1 × 16.2 × 14.4 mm in size. The internal echo pattern was heterogeneous, and lateral shadowing was observed. The echo was partially broken at the anterior margin but unbroken at the posterior margin (Fig. 1b). Subsequently, a needle biopsy was performed. Although proliferation of neoplastic spindle cells was suspected, no definitive diagnosis was obtained. Because the imaging studies and needle biopsy could not exclude the possibility of a malignant tumor, examination of the entire tumor was considered necessary, and an excisional biopsy was consequently performed. According to the macroscopic findings (Fig. 2), the cut surfaces appeared solid and milky white, and the tumor was clearly demarcated from the surrounding adipose tissue. According to the histopathological findings (Fig. 3), the tumor was a nodular lesion composed of spindle cells with deposition of abundant elastic fibers. It was not encapsulated, and it proliferated in the mammary tissue and partially extended into the adipose tissue. Although mitotic activity was relatively high at 10 mitoses/20 high-power fields, the Ki-67 level was only 3%. No apparent malignant changes were observed. The spindle cells were positive for α-smooth muscle actin (α-SMA) and vimentin but negative for pancytokeratin, cytokeratin 14, estrogen receptor, progesterone receptor, β-catenin, cluster of differentiation 34 (CD34), desmin, S100, and synaptophysin. These findings led to a diagnosis of NF. The resection margin was tumor-free. At the latest follow-up, 12 months postsurgery, no recurrence was observed.

## Discussion

We present a case of an elderly female with NF of the breast. While the mean age of patients in a previously reported study was 39 years (range, 17–84 years) [1], our patient is the oldest among the reported patients with NF of the breast, at 88 years. NF is a proliferative lesion of reactive fibroblastic cells arising from the fascia of the subcutaneous tissue. This lesion can occur anywhere in the body but is rarely found in the breast [2]. Nonetheless, NF of the breast is the first histological type of mesenchymal tumor added to the World Health Organization classification in 2012 [3]; as the awareness of this disease increases in the future, more patients may consequently be diagnosed with it.

The awareness of NF of the breast is important because, despite its benign nature, this disease resembles breast cancer in terms of its clinical course and imaging findings, and because the diagnostic rate by biopsy is low. In other words, the factors raising suspicion of malignancy in this disease include relatively rapid growth over 3 to 4 months [3], mammographic findings of spiculation and distortion, and ultrasound findings of a hypoechoic mass with irregular margins and a high depth-width ratio [4]. Because the contrast-enhanced magnetic resonance mammography findings reportedly differ among the NF subtypes [5], this imaging modality is less useful for differentiation between benign and malignant lesions. A possible reason why imaging studies, particularly mammography and ultrasonography, yield breast cancer-like findings is that they reflect the morphological development of non-encapsulated tumors invading the adipose tissue.

Preoperative diagnosis is difficult to establish by fine-needle aspiration cytology (FNA) or core needle biopsy. Although there is one report on NF successfully diagnosed by FNA, which yielded samples containing abundant homogeneous spindle fibroblastic cells against the background of inflammatory cells and mucoid substances [6], the number of cases of NF diagnosed by FNA is small [7]. Because NF morphologically resembles spindle cell carcinoma, excisional biopsy is recommended [2].

The differential diagnoses of NF include lesions mainly characterized by proliferation of spindle cells; especially, spindle cell carcinoma and sarcoma among malignant lesions, and fibromatosis and myofibroblastoma among benign lesions, morphologically resemble NF and need to be differentiated. Although no doubt exists regarding the importance of morphological confirmation of the diagnosis, immunostaining is a useful auxiliary means. The characteristic immunostaining patterns of NF are positivity for α-SMA and myositis-specific autoantibodies and negativity for β-catenin, CD34, and cytokeratin [2]. However, attention should be paid to cytokeratin, because one report stated that 9% of cases are in fact positive for cytokeratin [8]. In our case, NF was diagnosed based on the immunostaining results (positivity for α-SMA and negativity for β-catenin, CD34, and cytokeratin) in addition to the morphological findings.

Although excisional biopsy is unnecessary in cases with appropriate diagnosis, it is frequently performed to exclude the possibility of malignancy. Cases following various clinical courses have been reported, including cases of NF growing after FNA [9] and cases exhibiting substantial regression of lesions after core needle biopsy [7]. However, recurrence at the same site has not yet been reported. Excisional biopsy is considered an essential and sufficient procedure [2], and accurate diagnostic methods are thus required to prevent over-diagnosis.

## Conclusions

In conclusion, although NF occurring in the breast of very old female, as seen in our case, is rare, caution should be exercised, because NF shows rapid growth and clinically resembles malignant tumors. Hence, we recommend including NF as one of the differential diagnoses if biopsy samples show proliferation of spindle tumor cells. Confusion with this pathology and neoplasm can be very dramatic; pathologist must be very aware to make diagnosis of cancer.

## Abbreviations

CD34: Cluster of differentiation 34
FNA: Fine-needle aspiration
NF: Nodular fasciitis
α-SMA: α-Smooth muscle actin
